# Impact of Dexamethasone on the Pathogen Profile of Critically Ill COVID-19 Patients

**DOI:** 10.3390/v15051076

**Published:** 2023-04-28

**Authors:** Cathrin Kodde, Finja Timmen, Sven Hohenstein, Andreas Bollmann, Marzia Bonsignore, Ralf Kuhlen, Irit Nachtigall, Selcuk Tasci

**Affiliations:** 1 Germany Department of Respiratory Diseases, Lungenklinik Heckeshorn, Helios Hospital Emil-von-Behring, 14165 Berlin, Germany; 2 Department of Infectious Diseases and Respiratory Medicine, Charité—Universitaetsmedizin Berlin, 13353 Berlin, Germany; 3 Medical Faculty, University of Bonn, 53113 Bonn, Germany; 4 Department of Cardiology, Heart Center Leipzig at University of Leipzig, 04289 Leipzig, Germany; 5 Division of Infectious Diseases and Prevention, Helios Hospitals, 47166 Duisburg, Germany; 6 Center for Clinical and Translational Research, Helios Universitätsklinikum Wuppertal, University of Witten/Herdecke, 42283 Wuppertal, Germany; 7 Helios Health, 10117 Berlin, Germany; 8 Division of Infectious Diseases and Infection Prevention, Helios Hospital Emil-von-Behring, 14165 Berlin, Germany; 9 Institute of Hygiene and Environmental Medicine, Charité—Universitaetsmedizin Berlin, 12203 Berlin, Germany; 10 Department of Respiratory Diseases, Helios Hospital Bonn/Siegburg, 53721 Siegburg, Germany

**Keywords:** SARS-CoV-2, critical care, bacteria, COVID-19 pandemic

## Abstract

Background: Even though several therapeutic options are available, COVID-19 is still lacking a specific treatment regimen. One potential option is dexamethasone, which has been established since the early beginnings of the pandemic. The aim of this study was to determine its effects on the microbiological findings in critically ill COVID-19 patients. Methods: A multi-center, retrospective study was conducted, in which all the adult patients who had a laboratory-confirmed (PCR) SARS-CoV-2 infection and were treated on intensive care units in one of twenty hospitals of the German Helios network between February 2020–March 2021 were included. Two cohorts were formed: patients who received dexamethasone and those who did not, followed by two subgroups according to the application of oxygen: invasive vs. non-invasive. Results: The study population consisted of 1.776 patients, 1070 of whom received dexamethasone, and 517 (48.3%) patients with dexamethasone were mechanically ventilated, compared to 350 (49.6%) without dexamethasone. Ventilated patients with dexamethasone were more likely to have any pathogen detection than those without (*p* < 0.026; OR = 1.41; 95% CI 1.04–1.91). A significantly higher risk for the respiratory detection of *Klebsiella spp.* (*p* = 0.016; OR = 1.68 95% CI 1.10–2.57) and for *Enterobacterales* (*p* = 0.008; OR = 1.57; 95% CI 1.12–2.19) was found for the dexamethasone cohort. Invasive ventilation was an independent risk factor for in-hospital mortality (*p* < 0.01; OR = 6.39; 95% CI 4.71–8.66). This risk increased significantly in patients aged 80 years or older by 3.3-fold (*p* < 0.01; OR = 3.3; 95% CI 2.02–5.37) when receiving dexamethasone. Conclusion: Our results show that the decision to treat COVID-19 patients with dexamethasone should be a matter of careful consideration as it involves risks and bacterial shifts.

## 1. Introduction

When the first COVID-19 cases appeared in early 2020, treatment options were limited. The novel acute respiratory syndrome coronavirus 2 (SARS-CoV-2) was associated with flu-like symptoms, but also with various complications including viral pneumonia, acute respiratory distress syndrome (ARDS), thromboembolic events, and bacterial co- and superinfections. Up to this date, there is no specific treatment for COVID-19 [1]. Several therapeutic options exist in mild-to-moderate cases of COVID-19 for patients who have a high risk of a severe outcome. These include antiviral drugs [2], anti-SARS-CoV-2 monoclonal antibodies, and immunomodulatory therapeutics that help the immune system recognize and fight SARS-CoV-2 [3,4]. With the occurrence of new variants, certain therapeutic options have already become less effective [5].

In the early beginnings of the pandemic, the international guidelines recommended the prophylactic use of antibiotics to prevent and treat bacterial pneumonia in hospital-treated COVID-19 patients. However, studies have shown that the extensive use of antibiotics led to more pathogen detection, especially those with antimicrobial resistance [6,7,8]. Not only did antibiotics play a role during the COVID-19 pandemic; alternative therapeutic options, including Traditional Chinese Medicine (TCM), also played a key role. Studies showed that certain herbs used in TCM helped to relieve COVID-19 symptoms, such as a cough and a fever [1].

From July 2020, corticosteroids became the state-of-art in the treatment of severe COVID-19 because a decrease in the 28 days mortality of critically ill COVID-19 patients was reported [9]. The time of administration is crucial, as corticosteroids are known for their anti-inflammatory and immunomodulatory effects. A known life-threatening complication of an infection with SARS-CoV-2 is the cytokine storm, which induces the excessive production and circulation of pro-inflammatory markers in the human body similar to those observed in septic patients [10]. Corticosteroids can reduce this excessive host inflammatory response and suppress progression. As a side effect, corticosteroids impair the innate-immune-system-mediated pathogen clearance. This is one of the reasons why corticosteroids may have an adverse outcome in the beginning of an infection, as they compromise the patient’s innate immune system, leading to a more severe clinical course [11,12].

During the second COVID wave, studies emerged that showed a decrease in in-hospital mortality for critically ill patients receiving dexamethasone, which resulted in better clinical outcomes [9,13]. However, as the COVID-19 pandemic progressed, studies showed an increase in bacterial and fungal co- and superinfections [7,8]. The reason for this is still subject to investigation, but a correlation with the application of corticosteroids and the extensive use of antibiotics has been assumed [6,14,15,16]. In particular, patients in intensive care units (ICUs) are at a higher risk of nosocomial infections and fungal/bacterial co- and superinfections, as they require multiple invasive procedures, such as the insertion of central venous catheters or arterial lines and invasive mechanical ventilation.

The aim of the study was to determine the effect of dexamethasone on microbiological findings in critically ill COVID-19 patients, with a focus on invasive mechanical ventilation.

## 2. Material and Methods

### 2.1. Study Design

In this multi-center, observational, and retrospective study, all patients above 18 years of age were included who had a laboratory-confirmed (PCR) SARS-CoV-2 infection and were treated for or with COVID-19 in the ICU. The SARS-CoV-2 infection must have been detected before or within 7 days after admission to the ICU and the duration had to be 24 h or more.

Data were collected from 20 hospitals which had the highest number of COVID-19 patients on ICU within the German Helios network between February 2020 and March 2021 and then aligned with corresponding claims data through an anonymized data transfer.

The data of the microbiological findings came from iNOK, the surveillance program of the Helios hospitals. iNOK is a surveillance database that captures community- and hospital-acquired pathogens and other infectious diseases within the hospitals.

Microbiological data records include bacterial/fungal organism genus and localization of detection. The samples were collected when a co- or superinfection was suspected through blood cultures (BS), or through sputum and lower respiratory tract samples (RS), including bronchoalveolar lavage fluid and tracheobronchial aspirate.

Positive microbiological results of superficial screening swabs and urine samples were excluded as they did not allow an interpretation of their clinical relevance and were not considered clinically relevant as COVID-19-related infections. To determine possible risk factors for a bacterial shift, a cohort of patients was formed who received dexamethasone at any point of their treatment. Dexamethasone treatment was applied as according to the national guidelines. Patients receiving hydrocortisone were excluded to ensure comparability. Generated by the claims data, two groups were formed according to their application of oxygen: invasive ventilation (mechanical ventilation, extracorporeal membrane oxygenation; ECMO) and non-invasive ventilation (none, nasal cannula = low-flow oxygen, high-flow oxygen, or non-invasive ventilation: NIV). Those who at first had *other* types of ventilation but then required invasive ventilation were categorized into the group with invasive ventilation. Due to the difficulties in collecting the respiratory specimens (RS) in non-intubated patients, a significantly higher number of these samples were obtained in invasively ventilated patients. Therefore, further investigations into the pathogen profile of RS were only conducted in invasively ventilated patients.

Cases with discharge due to hospital transfer or an unspecified reason were excluded for the analysis of in-hospital mortality.

### 2.2. Ethical Consent

The study was approved by the Ethics Committee of the Medical Faculty University Leipzig (490/20-EK) and registered in the German Clinical Trials Register (DRKS 00027266). The study was supported by the Helios research grant HCRI ID 2021-0339.

### 2.3. Statistical Analysis

Inferential statistics were based on generalized linear mixed models (GLMMs) specifying hospitals as a random factor. We employed the logistic GLMMs function for dichotomous data and LMMs for continuous data. Count data were analyzed with Poisson GLMMs. Effects were estimated with the lme4 package (version 1.1-26) in the R environment for statistical computing (version 4.0.2, 64-bit build). For all tests, a two-tailed 5% error criterion was applied for significance.

For the description of the patient characteristics of all the cohorts, a χ^2^-test was employed for binary variables and an analysis of variance for numeric variables. Proportions, means, standard deviations, and *p*-values are shown.

For the comparison of proportions of symptoms as well as selected treatments and outcomes in the different cohorts, a logistic GLMM was used with the logit link function. Proportions and odds ratios are reported together with confidence intervals and *p*-values.

Means, standard deviations, medians, interquartile ranges, and *p*-values are shown. The computation of *p*-values for the continuous dependent variables is based on the Satterthwaite approximation for degrees of freedom. Mortality data were analyzed using multivariable models. Independent variables were male sex, age, Elixhauser Comorbidity Index (ECI), dexamethasone, and invasive ventilation.

Statistics are reported for ECI as well as its items. For the weighted ECI, the AHRQ algorithm was applied.

## 3. Results

During the study period, a total number of 1.776 patients were included. From this group, 1070 patients received dexamethasone at any point of their treatment, and 706 patients did not receive dexamethasone. Baseline characteristics are shown in Table 1. In the dexamethasone group, 517 (48.3%) patients were mechanically ventilated, compared to 350 (49.6%) in the group without dexamethasone. Sixty-one patients were treated with hydrocortisone but were excluded to ensure comparability. One patient received lopinavir/ritonavir. Remdesivir was administered in 100 patients. There is a male predominance in both groups (dexamethasone n = 710/66.4%; no dexamethasone n = 437/62%). The most common comorbidities were hypertension (62.8%), diabetes (43.3%), and obesity (22.2%), but also COVID-19 disease-related comorbidities, including electrolyte disorders (64.75%), cardiac arrhythmias (37.05%), renal failure (33%), and congestive heart failure (32.5%). Patients with dexamethasone had a lower Elixhauser Comorbidity Index (ECI).

Dexamethasone-treated patients had a significantly longer median length of stay in the ICU, irrespective of whether they were ventilated (16.0 vs. 14.0; *p* < 0.001) or not (6.0 vs. 4.0 days; *p* < 0.001). In addition, they were significantly mechanically ventilated for longer than those without dexamethasone (304.0 versus 268.0 h; *p* < 0.001).

A total of 870 microbiological pathogens were detected in 510 patients with an average of 1.71 findings per person. Of the 1070 patients who underwent dexamethasone therapy, 325 patients (30.4%) were recorded as having at least one microbiological finding, compared to 185 (26.2%) patients in the non-dexamethasone group (*p* = 0.116).

In the invasively ventilated patients (n = 867), a total of 750 samples positive for pathogens were detected (287 BS; 463 RS). Every second patient had a detection of a pathogen (n = 412; 47.5%) with an average of 1.82 findings per person. The most frequently detected pathogens in blood cultures in the ventilated cohort were as follows (in descending order): *Staphylococcaceae*, *S. aureus*, *Candida spp.*, *Enterobacterales*, *E. coli*, and *Pseudomonas aeruginosa*. Detected pathogens in the respiratory tract were as follows (in descending order): *Klebsiella spp.*, *S. aureus*, *Pseudomonas aeruginosa*, and *E. coli* (see Table 2, “Pathogen detection of ventilated patients”).

In general, ventilated patients with dexamethasone were more likely to have a pathogen detected than those without dexamethasone (*p* < 0.026; odds ratio (OR) 1.41; 95% CI 1.04–1.91). Patients who were mechanically ventilated and received dexamethasone had an average bloodstream detection rate of 0.35 pathogens per patient, compared to 0.30 pathogens per patient without dexamethasone (*p* < 0.231; OR = 0.84; 95% CI 0.64–1.12). Patients with dexamethasone had an average respiratory tract pathogen detection of 0.59, in comparison to 0.45 without dexamethasone (*p* < 0.109; OR = 0.74; 95% CI 0.51–1.07).

For the entire group of *Enterobacterales*, including *Enterobacter spp.*, *E. coli*, *Klebsiella spp.*, *Proteus mirabilis*, *Citrobacter spp.*, and *Serratiae marcescens*, there was a significant increase in respiratory tract pathogen yield when dexamethasone was administered. One hundred and forty-eight of the five hundred and seventeen invasively ventilated patients with dexamethasone therapy (28.6%) had a pathogen of this group recorded, compared to 20.6% of ventilated patients without dexamethasone (*p* = 0.008; OR = 1.57; 95% CI 1.12–2.19).

**Figure 1:**
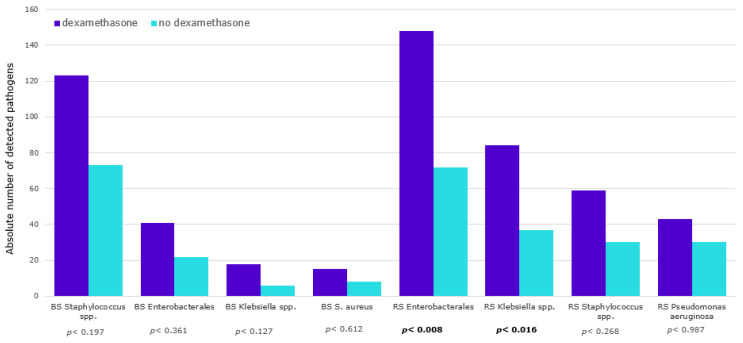
Pathogen detection in the invasive ventilation group. BS, blood samples; RS, respiratory samples; bold indicates statistically significant.

A significantly higher risk for respiratory detection of *Klebsiella spp.* was found for dexamethasone-treated patients (*p* = 0.016; OR = 1.68; 95% 1.10–2.57) (see Figure 1).

Mortality analysis was based on 1526 patients due to the exclusion of cases with discharge due to hospital transfer or for an unspecified reason. The overall mortality rate was 52.2% (796/1526), and 62.2% (495/796) died in the dexamethasone group. From 844 patients without invasive ventilation, 306 (36.3%) died, while 196 (64%) of them were treated with dexamethasone. In the invasively ventilated group (n = 682), we observed a mortality rate of 71.8% (490/682), and 299 patients (61%) out of this group were treated with dexamethasone.

Using a multivariable analysis, we identified invasive ventilation as an independent risk factor for in-hospital mortality. In addition, comorbidities, measured by the ECI, and age had a significant influence on the mortality in the general cohort (see Table 3). We did not detect any correlation between the dexamethasone therapy and in-hospital mortality for those younger than 80 years of age. However, the odds for in-hospital mortality in patients aged 80 years or older were 3.3-fold higher (*p* < 0.01; OR = 3.3; 95% CI 2.02–5.37) when receiving dexamethasone treatment.

## 4. Discussion

Dexamethasone is part of the standard therapy for severely or critically ill COVID-19 patients since the middle of 2020. Dexamethasone is seen as a cornerstone in the treatment of severe COVID-19 cases, and its appropriate use has led to a lowered 28-day mortality, as well as shortening the duration of ventilation, leading to favorable clinical outcomes for patients [9,17].

Even though early studies have stated that dexamethasone has a positive effect on mortality and clinical outcomes, other publications now suggest a more careful use as co-infections are on the rise [9,11,17]. In this retrospective cohort study that compared the incidences of bacterial and fungal pathogen detection in ICU patients, one of the key findings was that dexamethasone therapy in mechanically ventilated patients is a significant risk factor for pathogen detection.

These results show that the decision to treat COVID-19 patients with dexamethasone should always be a matter of careful consideration as it also involves risks. This dichotomy is confirmed by other studies that showed an association between corticoid therapy and an increased incidence of bacteremia [15,18,19,20]. Rothe et al. described a significant increase in polymicrobial detection, an association with respiratory infection complications, and a higher mortality rate in the group of invasively ventilated patients treated with dexamethasone (49.6% of invasively ventilated patients without dexamethasone vs. 55.9% of invasively ventilated patients with dexamethasone) [18]. When the therapy is appropriately applied, namely in patients at an imminent risk of cytokine storm, the anti-inflammatory and immunomodulatory effects of dexamethasone work in favor of the severely ill COVID-19 patients, reducing both the need of mechanical respiratory assistance as well as the mortality rate.

Nevertheless, the administration of corticosteroids generally increases the risk of serious infection, as well as colonization by fungal and bacterial pathogens [12,18]. In particular, ICU patients are at a significantly higher risk due to their compromised initial condition, the use of invasive devices, and exposition to, for instance, nosocomial pathogens. Our results point in the same direction. We showed a significant association of dexamethasone with both the general incidence of pathogens as well as individual pathogens.

In the present study, pathogens were detected in nearly 30% of all patients, either in respiratory samples or in bloodstream samples. Within the invasively ventilated patients, almost 50% were sample-positive for any pathogen, which is a slightly higher incidence compared to other studies [18,19]. This discrepancy may result from the difficulties in correctly detecting bacterial infections. Pathogens are often not detected in cultures because an empiric antibacterial therapy would have already taken place before, and contamination can also occur. A more precise analysis could be possible with a prospective study design that considers proven clinical infections.

Furthermore, we saw that invasively ventilated patients were at a higher risk for the detection of bacteria compared to non-ventilated patients. This is in accordance with the observation of higher rates of ventilator-associated pneumonia (VAP) and co-infections, especially bloodstream infections, often caused by multidrug-resistant bacteria, in this patient group [21,22].

Within the invasively ventilated group in this study, dexamethasone increased the risk for the respiratory detection of *Klebsiella spp.* and *Enterobacterales*. Prior studies also found a bacterial shift due to dexamethasone. However, a higher detection rate of *S. aureus*, *E. faecalis*, and *Enterobacterales* was found there [18].

The latest studies show that the number of deaths and severe clinical courses decreased thanks to the stabilization of resources, dynamics of the viral variations, and further development of therapies and vaccines [23,24]. However, we observed an overall mortality rate of 52.2% during our study period from January 2020 to March 2021. Furthermore, a high mortality rate of 71.8% among the invasively ventilated patients was seen. The ICU mortality rate ranges from 38% to 75%, with the highest rate being for those who were mechanically ventilated [14,25,26]. The high mortality found in this study could be due to the fact that the patient population is older compared to other studies with a different average age of the patients included. A meta-analysis examining 7 studies observed a median age of 60 years with an overall mortality rate of 32% [19]. A study by Rothe et al. saw a mortality rate of 55.9% in invasively ventilated patients treated with dexamethasone and a median age of 68 years [18,25].

Using a multivariable analysis, invasive ventilation was identified as an independent risk factor for in-hospital mortality. In addition, comorbidities measured by the ECI, and age, had significant influences on the mortality in the general cohort. We did not detect any association between dexamethasone therapy and in-hospital mortality for individuals younger than 80 years of age. However, the odds of in-hospital mortality were 3.3-fold in patients aged 80 years or older when receiving dexamethasone treatment. This, is in accordance with the recovery trial, which showed an advantage for dexamethasone only in oxygenated and ventilated patients younger than 70 years [9].

Crothers et al. reported a disadvantage of dexamethasone in patients not requiring oxygen in terms of 90-day mortality [27]. However, the exact reason for this association remains unclear. One assumption is that dexamethasone may suppress the immune system in patients who do not require oxygen, thus leading to secondary infections. Additionally, dexamethasone may also lead to fluid retention and an elevated blood sugar level, which may result in a worsened clinical outcome for some COVID-19 patients [27]. Regarding the increased mortality risk in the oldest age group with dexamethasone, further research should be conducted, and an adjustment for the guidelines should be considered.

### Strengths and Limitations

Due to the retrospective observational study design, there is no differentiation between the detection of pathogens and clinically relevant infections, especially secondary pneumonia. Furthermore, not every patient had a microbiological specimen taken. This limitation must be kept in mind when interpreting the pathogen evidence. Furthermore, clinical characteristics of the study population, such as vaccination status or the severity of disease (e.g., laboratory parameters and radiological findings), have not been examined.

The ratio of treatment with dexamethasone to no treatment with dexamethasone is 3:2. Dexamethasone treatment for critically ill COVID-19 patients in the ICU was established in Germany during the second wave and resulted in an overall higher number of patients treated with dexamethasone. Since it is not a prospective study design, there may be a bias towards the possible delayed implementation of guidelines (published spring 2020), and the recommended use of dexamethasone when oxygen supply is mandatory means that severely ill patients were more likely to receive dexamethasone. Another limiting factor is that other therapeutic strategies such as oxygenation and antiviral treatment have also changed significantly in addition to the establishment of dexamethasone. Earlier in the pandemic, rapid invasive mechanical ventilation was recommended. However, as the pandemic progressed, non-invasive ventilation and other lung-protective measures became important. These additional influential factors may have contributed to a bias in the study.

Regarding the statistical analysis, the discriminatory power of the significance tests is considered to be a statistically limiting factor due to the large amount of data and the associated sub-analysis, which possibly reduced the discriminatory power.

A clear benefit of the study is that the findings can be extrapolated as our study took place in different hospitals, ranging from small specialized hospitals to tertiary care centers. From the beginning of the pandemic, internal advisors and guidelines were available regarding the handling of severely ill COVID-19 patients in the ICU as well as the handling of suspected bacterial findings, based on the respective state of knowledge, and were continually adjusted.

Another strength of our study is its focus on ICU-treated patients. Thus, we analyzed the effects of dexamethasone in COVID patients only, with an indication for the use of dexamethasone in either ventilated or non-ventilated patients.

## 5. Conclusions

In summary, the use of dexamethasone for critically ill COVID-19 patients was established and widespread during the pandemic. Nevertheless, it is important to note that its extensive use may also involve risks, which are demonstrated by a higher incidence of pathogen detection, especially in mechanically ventilated patients. Additionally, the use of dexamethasone was associated with a higher in-hospital mortality in patients who were older than 80 years of age. Further research needs to be conducted and guidelines should be adjusted regularly.

## Figures and Tables

**Table 1:** Baseline characteristics of the study population.

	Dexamethasone n = 1070		No Dexamethasone n = 706		*p*-Value Dexamethasone vs. No Dexamethasone	
	LF/HF-O2, NIV n = 553	Invasive Ventilation/ ECMO n = 517	LF/HF-O2, NIV n = 356	Invasive Ventilation/ ECMO n = 350	LF/HF-O2, NIV	Invasive Ventilation/ ECMO
Age						
Mean (SD)	69.3 ± 15.2	67.6 ± 11.8	71.1 ± 14.6	69.5 ± 11.9	0.073	0.018
Sex						
Female–Male	202:351 (11.4%:19.8%)	158:359 (8.9%:20.2%)	157:199 (8.8%:11.2%)	112:238 (6.3%:13.4%)	0.027	0.708
Elixhauser Comorbidity Index (AHRQ algorithm, 30 comorbidities)						
Mean (SD)	13.0 ± 10.6	17.2 ± 11.2	15.3 ± 12.6	20.8 ± 12.0	0.003	<0.001
Pathogen Samples						
Total n = 870	74	486	46	264		
Affected patients n = 510 (28.7%)	60 (11.8%)	265 (52.0%)	38 (7.5%)	147 (28.8%)	0.999	0.015
Blood stream detections (40.8%)	42 (4.8%)	182 (20.9%)	26 (3.0%)	105 (12.1%)	0.491	0.231
Respiratory tract detections (59.8%)	32 (3.7%)	304 (34.9%)	20 (2.3%)	159 (18.3%)	0.956	0.109
Length of stay in ICU Median (ICR)	6 (3, 9)	16 (10, 26)	4 (2, 9)	14 (7, 24)	<0.001	<0.001
Duration of mechanical ventilation Median (ICR)	62 (24, 136)	304 (150, 538)	63 (17, 195)	268 (112, 473)	0.873	<0.001

**Table 2:** Pathogen profile of mechanically ventilated patients, blood stream samples (BS), and respiratory tract samples (RS).

	Proportion (n)	Odds Ratio (95% CI)	*p*-Value
BS *Enterobacterales*			
No dexamethasone	6.3% (22)		
Dexamethasone	7.9% (41)	1.28 (0.75–2.20)	0.361
BS *E. coli*			
No dexamethasone	1.4% (5)		
Dexamethasone	1.5% (8)	1.08 (0.35–3.34)	0.888
BS *Enterobacter spp.*			
No dexamethasone	2.6% (9)		
Dexamethasone	1.7% (9)	0.77 (0.29–2.01)	0.590
BS *Klebsiella spp.*			
No dexamethasone	1.7% (6)		
Dexamethasone	3.5% (18)	2.07 (0.81–5.26)	0.127
BS *Pseudomonas aeruginosa*			
No dexamethasone	1.4% (5)		
Dexamethasone	1.9% (10)	1.46 (0.48–4.43)	0.507
BS *Staphylococcaceae*			
No dexamethasone	20.9% (73)		
Dexamethasone	23.8% (123)	1.26 (0.89–1.79)	0.197
BS *S. aureus*			
No dexamethasone	2.3% (8)		
Dexamethasone	2.9% (15)	1.26 (0.52–3.05)	0.612
RS *Enterobacterales*			
No dexamethasone	20.6% (72)		
Dexamethasone	28.6% (148)	1.57 (1.12–2.19)	0.008
RS *E. coli*			
No dexamethasone	5.7% (20)		
Dexamethasone	8.9% (46)	1.58 (0.91–2.73)	0.102
RS *Enterobacter spp.*			
No dexamethasone	4.0% (14)		
Dexamethasone	5.0% (26)	1.26 (0.65–2.46)	0.494
RS *Klebsiella spp.*			
No dexamethasone	10.6% (37)		
Dexamethasone	16.2% (84)	1.68 (1.10–2.57)	0.016
RS *Pseudomonas aeruginosa*			
No dexamethasone	8.6% (30)		
Dexamethasone	8.3% (43)	1.00 (0.61–1.65)	0.987
RS *Staphylococcaceae*			
No dexamethasone	8.6% (30)		
Dexamethasone	11.4% (59)	1.30 (0.82–2.08)	0.268
RS *S. aureus*			
No dexamethasone	7.7% (27)		
Dexamethasone	10.6% (55)	1.35 (0.83–2.20)	0.229
Any Pathogen			
No dexamethasone	60.0% (210)		
Dexamethasone	68.1% (352)	1.41 (1.04–1.91)	0.026

Subgroups: *Enterobacterales* (*Enterobacter spp.*, *E. coli*, *Klebsiella spp.*, *Proteus mirabilis*, *Citrobacter spp.*, *Serratiae marcescens*), *Staphylococcaceae* (Staph. Aureus, coagulase negative *Staph. spp.*) Most detected single pathogens: *E. coli*, *Enterobacter spp.*, *Klebsiella spp.*, *Pseudomonas aeruginosa*, and *S. aureus*).

**Table 3:** Results of multivariable analyses of in-hospital mortality.

Variable	Odds Ratio (95% CI) Total Cohort Patients ≥ 80 Years		*p*-Value Total Cohort Patients ≥ 80 Years	
Male sex	0.98 (0.76–1.28)	1.29 (0.81–2.06)	0.91	0.28
Age	1.09 (1.08–1.10)	1.14 (1.06–1.23)	<0.01	<0.01
Elixhauser Comorbidity Index	1.04 (1.03–1.05)	1.01 (0.99–1.03)	<0.01	0.28
Dexamethasone	1.65 (1.27–2.14)	3.29 (2.02–5.37)	<0.01	<0.01
Invasive ventilation	6.39 (4.71–8.66)	4.42 (2.46–7.93)	<0.01	<0.01
Any pathogen	1.13 (0.85–1.50)	Not available	0.40	Not available

## Data Availability

Not applicable.
